# Differential survivorship of congeneric ornamental fishes under forecasted climate changes are related to anaerobic potential

**DOI:** 10.1590/1678-4685-GMB-2017-0016

**Published:** 2018-02-19

**Authors:** Luciana Mara Fé Gonçalves, Maria de Nazaré Paula da Silva, Adalberto Luis Val, Vera Maria Fonseca de Almeida-Val

**Affiliations:** 1 Laboratório de Ecofisiologia e Evolução Molecular, Instituto Nacional de Pesquisas da Amazônia (LEEM-INPA), Av. André Araújo, 2936; Petrópolis. 69067-375, Manaus, AM, Brazil.; 2 Programa de Pós-Graduação em Aquicultura, Universidade Nilton Lins, Av. Professor Nilton Lins, 3259; Parque das Laranjeiras 69058-030, Manaus, AM, Brazil.

**Keywords:** ornamental fish, enzyme activity, relative gene expression, IPCC scenarios, Lactate Dehydrogenase (Ldh)

## Abstract

Two Amazonian closely related tetras – cardinal *Paracheirodon axelrodi* and green neon *P. simulans* – were artificially acclimatized to environmental chambers mimicking future climate change scenarios (mild, moderate and extreme), using a microcosm facility. *P. simulans* survived (100%) to all scenarios after 30 days exposure, while *P. axelrodi* presented decreasing survival percentages according to environmental severity. These differences may be the reflection of distinct natural acclimatization to microhabitats between the species, which differ in thermal conditions. Survival responses might be related to differences in relative gene expression of lactate dehydrogenase (Ldh), suggesting that *P. axelrodi* anaerobic potential is lower or non-existent compared to *P. simulans*, not tolerating long-term thermal challenges. Accordingly, increases in temperature and in CO_2_ levels caused increases in energy demand and resulted in activation of the anaerobic pathway, as demonstrated by the higher enzyme levels measured in head and tail portions of both species. Sustained anaerobic glycolysis is possible when fish live in challenging environments (low oxygen or high temperature). Our results clearly show that *P. simulans* has a larger scope for survival to higher energy demands due to its increased anaerobic potential compared to *P. axelrodi.*

## Introduction

The review on lactate dehydrogenase (Ldh) tissue distribution in 245 fish species by Almeida-Val and Val (1993) suggested that hypoxia adaptation could be due to (i) predominance of isoform B_4_ in aerobic tissues, indicating permanent aerobic metabolism in tissues like heart and liver, and (ii) suppression of oxidative metabolism plus activation of anaerobic glycolysis, resulting in the predominance of isoform A_4_ in all tissues. The plasticity in regulating the expression of Ldh genes in fishes is one of the best biochemical adaptation processes to deal with oxygen and temperature environmental changes, besides the other ongoing impacts of climate change, as suggested by Hochachka and Somero (1973, 2002). According to the IVth report of the Intergovernmental Panel for Climate Change (IPCC, 2007), the global atmospheric concentration of greenhouse gases (carbon dioxide, methane and nitrous oxide) has increased since the Industrial Revolution. Notably, carbon dioxide (CO_2_) emissions increased from a pre-industrial level of approximately 280 parts per million (ppm) to over 400 ppm in 2016. Levels can increase to more than 800 ppm at the end of the 21st century, according Feely *et al.* (2004), reaching 1,250 ppm in an extreme scenario (IPCC, 2007). Changes in anthropogenic activities such as fossil fuel and land use have driven global warming. The consequences of such changes deserve attention, particularly regarding the effects of increased temperature and carbon dioxide levels in the tropics, including the Amazon (Nobre *et al.*, 2007, 2008).

Temperature, as a relevant environmental factor, can strongly affect fish physiology (Beitinger *et al.*, 2000). Under elevated temperatures, energy demand increases, requiring several metabolic adjustments from the organism, such as those described for fishes of the Amazon (Almeida-Val and Hochachka, 1995; Driedzic and Almeida-Val, 1996). In many cases, fishes respond to temperature rise by Ldh genes transcription and enzyme levels increase, elevating the anaerobic power to cope with cellular hypoxia caused by higher metabolic demands (Almeida-Val *et al.*, 2006; Heuton *et al.*, 2015). Furthermore, elevated CO_2_ concentrations tend to acidify the water (Guinotte and Fabry, 2008). Adverse short-term effects of high CO_2_ on fishes include respiratory and nervous system distress, imbalance of acid-base status, and changes in blood-O_2_ affinity (Ishimatsu *et al.*, 2004). In addition, long-term high CO_2_ exposure causes reduced growth rate, reproduction disorders and death (Ishimatsu *et al.*, 2005; Oliveira and Val, 2016).

Regional models of climate change indicate temperature increases between 2 and 6 °C in South America, as well as a decline in precipitation in eastern Amazonia (Ambrizzi *et al.*, 2007; Salazar *et al.*, 2007). Also, different climate models project a reduction of tropical forest cover, which might lead to a “savannization” in eastern Amazonia (Li *et al.*, 2006; Salazar *et al.*, 2007), affecting the biological conservation of terrestrial and aquatic ecosystems. Fish fauna diversity can be vulnerable to these challenges, particularly the commercially important, tiny ornamental fishes cardinal tetra (*Paracheirodon axelrodi*) (Schultz 1956) and green neon tetra (*Paracheirodon simulans*) (Géry 1963), analyzed in this study. *P. axelrodi* is the most exported Amazonian aquarium fishes (Anjos *et al.*, 2009), which retain high microsatellite genetic variability and low genetic structure, even though they are intensely collected by extractive fisheries (Beheregaray *et al.*, 2004; D’Assunção AAA, 2006, MSc. Thesis, Brazilian National Institute for Research of the Amazon, Manaus). These two congeneric species are endemic to the Amazon and occur in small streams that drain into Negro and Orinoco River basins (Axelrod *et al.*, 1986). As observed by Marshall *et al.* (2011), the two species inhabit palm swamps with similar physicochemical conditions, though they have specific thermal preferences: the habitat of *P. simulans* reaches 35 °C, while the maximum temperatures of the *P. axelrodi* habitat roughly reach 29 °C.

Therefore, considering the differential thermal niches occupied by these two ornamental tetras and the similarities they share due to their contiguous environments and phylogeny, we anticipate differential responses to the near-future impacts of ongoing climate change, which may result in different ecological threats. In the present work, we tested the influence of three different IPCC scenarios projected for the year 2100, based on the Special Report on Emission Scenarios (SRES), to both *P. axelrodi* and *P. simulans* to understand how they respond to forecasted climate change.

## Material and Methods

This study followed the Brazilian Guidelines from the National Board of Control and Care for Ethics in the use of Experimental Animals (CONCEA/MCTI) and was approved by the INPA’s Committee of Ethics on Animal Care (Protocol 024/2012). Voucher specimens were deposited at INPA’s Fish Collection (38.318 for *P. axelrodi* and 38.319 for *P. simulans*).

### Sampling and maintenance of fish

Adult specimens of *P. axelrodi* and *P. simulans* purchased in a local ornamental fish shop (Prestige Aquarium LTDA) were transported to the Laboratory of Ecophysiology and Molecular Evolution (CBIO/INPA) and kept indoors for 30 days in 150 L polystyrene tanks under constant aeration. The animals were fed *ad libitum* with commercial dry food pellets (35% protein content).

### Experimental setup: climate change simulations in microcosms

Both species were exposed to three climate scenarios foreseen by IPCC (2007), aiming to investigate the effects of climate change on fish’s survival and the activation of their anaerobic metabolism (Ldh gene expression and enzyme activity). Temperature, CO_2_ concentration, air humidity, and photoperiod were automatically controlled in environmental rooms (microcosms) under a real-time protocol (Dragan F, Gutierrez D, Oliveira A, Almeida-Val V and Val A, unpublished), according to three main scenarios: mild scenario or B1 (+1.5 °C and +200 ppm CO_2_ over the current scenario); moderate scenario or A1B (+2.5 °C and +400 ppm CO_2_ over the current scenario); and extreme scenario or A2 (+4.5 °C and +850 ppm CO_2_ over the current scenario). The control room mimics the temperature and CO_2_ levels of a pristine forest nearby the laboratory. A Proportional Integral Derivative system monitored and adjusted the environmental parameters every other minute in each microcosm based on the control room (current scenario). Light-dark cycle was set to 12:12h and humidity was set as derived condition.

### Exposure to forecasted climate scenarios for the year of 2100

Two hundred specimens of *P. axelrodi* (0.09 ± 0.004 g and 2.1 ± 0.03 cm) and *P. simulans* (0.06 ± 0.008 g and 1.7 ± 0.03 cm) were transferred to each of the eight plastic aquaria (18 L). Fish remained in aerated water and were incubated in the four microcosms as mentioned above (one aquarium per species per room). Average water temperature, pH, and dissolved oxygen were measured daily before transferring the aquaria of both species to the microcosms. For *P. axelrodi*, aquaria temperature, pH and dissolved oxygen were 26.63 ± 0.12 °C; pH 6.50 ± 0.38 and 6.39 ± 0.23 mgO_2_L^-1^, respectively. For *P. simulans*, water conditions were 26.67 ± 0.12 °C; pH 6.48 ± 0.20 and 5.81 ± 0.24 mgO_2_L^-1^.

After one-week artificial acclimatization, all experimental aquaria were transported to the baseline scenario (control room) and sequentially (each 48 h) transferred to the next microcosm with previously set climate scenarios. Fish were sampled at two and 30 days after being transferred to a given microcosm. For each exposure, 48 individuals of *P. axelrodi* and *P. simulans* (twelve fish per species, per climate scenario) were carefully collected using a sterile tweezer and immediately stored in liquid nitrogen until RNA extractions and enzyme assays. Ultra-rapid freezing of the animals by direct immersion in liquid nitrogen (-180 °C) was the physical euthanasia method performed according to the Brazilian CONCEA guidelines for minute ornamental fishes.

Each aquarium had water quality checked twice a day over the experimental period using a digital oxygen meter YSI (Yellow Springs Instruments) model 55/12 for temperature and dissolved oxygen, a digital pH-meter UltraBASIC UB-10 (Denver Instrument Co.), and a colorimetric method for carbon dioxide concentration (Boyd and Tucker, 1992). The water of aquaria was partially (50%) renewed every other day. Survival percentages were measured by counting fish with signs of pre-death, i.e., erratic swimming behavior or loss of equilibrium (LOE). These animals were removed from the experimental aquaria, euthanized and appropriately discarded.

### RNA extraction, cDNA synthesis, sequencing, and primer design

Whole fish from each tetra species (*n*=6) were homogenized in 500 μL of TRIzol Reagent (Life Technologies) according to the manufacturer’s instructions for total RNA extraction. We used a NanoDrop 2000 Spectrophotometer (Thermo Fisher Scientific) to check concentration and quality of extracted RNA, and electrophoresis on 1% agarose formaldehyde gel to verify RNA integrity. We used DNase I (Life Technologies) to degrade genomic DNA in RNA samples. Synthesis of cDNA was obtained by reverse transcription reaction using RevertAid H Minus First Strand cDNA Synthesis kit (Fermentas), following the manufacturer’s instructions. Partial sequences of *ldh-a, ldh-b*, and *18S* genes were obtained using primers previously designed from a conserved region of other teleost fishes available in the GenBank database. The *ldh-a, ldh-b*, and *18S* primer sequences were: 5’-GG(A/T) GCC CG(C/T) CAG CAG GA-3’ (forward) and 5’-ATG GCC CAG GA(G/A) GTG TAG CC-3’ (reverse); 5’-TGG GAG TGG GGC AAG TGG GC-3’ (forward) and 5’-ACT GTG TTT GAC GAT CTG AGG-3’ (reverse); and 5’-GGA ATG AGT ACA CTT TAA ATCC-3’ (forward) and 5’-GGG GCG CCG AGA GGC AGG GGC-3’ (reverse), respectively. All PCR products were sequenced with 1 μL of Big Dye fluorescent dye (Applied Biosystems) and run on an ABI 3130XL automatic DNA sequencer (Applied Biosystems). The acquired partial nucleic acid sequences (Table S1) were analyzed using the BLAST program at the National Center for Biotechnology Information (NCBI) website and then used to generate *P. axelrodi* and *P. simulans* specific qRT-PCR primers.

### Quantitative real-time PCR

RNA extraction from 96 individuals (48 *P. axelrodi* and 48 *P. simulans*) and the synthesis of the first strand cDNA followed the method of reverse transcription, as mentioned above. The *ldh-a* and *ldh-b* relative gene expression were assessed by quantitative real-time PCR (qRT-PCR) on an ABI Prism 7500 sequence detection system (Applied Biosystems). Table 1 shows the primer pairs for all genes from both species, which were designed using Oligo Explorer software version 1.1.2 (free software developed by Teemu Kuulasmaa). Real-time PCR reactions were performed using 1 μL of cDNA (concentration of 1μg), 1 μL of each primer (concentration 2.5 pM), 2 μL of nuclease-free water (Life Technologies) and 5 μL of SYBR Green PCR Master Mix (Applied Biosystems) in a total volume of 10 μL. The following conditions were used: 2 min at 50 °C and an initial denaturation step at 95 °C for 10 min, followed by 40 cycles of 95 °C for 15 s, and 60 °C for 1 min (annealing temperature of all primers). Melting curve analyses done after running the PCR protocol confirmed the presence of a single product-specific melting temperature, as follows*: P. axelrodi* – *ldh-a*, 76.8 °C; *ldh-b*, 78.8 °C and *18S*, 81.1 °C; and *P. simulans* – *ldh-a*, 77.1 °C; *ldh-b*, 81.2 °C and *18S*, 81.5 °C. PCR amplification efficiency for each primer set was calculated by serial dilution curves obtained from a pool of experimental samples (1 to 0.001 μg cDNA concentration; *n*=6). All primer pairs showed optimal PCR efficiency: 2.0 for *ldh-a* and *18S* and 1.99 for *ldh-b* of *P. axelrodi;* and 1.95 for *ldh-a*, 1.98 for *18S* and 1.97 for *ldh-b* for *P. simulans*, as well as a high Pearson correlation coefficient (r > 0.95).

**Table 1:** Quantitative real-time PCR primer sets for *Paracheirodon axelrodi* and *P. simulans.*

Gene	Forward primer (5’-3’)	Reverse primer (5’-3’)	Length (bp)	Amplicon length (bp)
*P. axelrodi*				
*ldh-a*	TCAGATCGTCAAGTACAGCC	AACTTCCAGGTGACGTAGGT	20	84
*ldh-b*	TAGTCCTTGTCAGCCACGAT	AGGGACTTGTGTGATGAGCT	20	126
*18S*	GGAACCCAAAGACTCTGGT	TAATCAAGAACGAAAGTCGG	19	144
*P. simulans*				
*ldh-a*	TAACGGGTACATCTTGGGAG	GGCTAACTCCAGCAACGTTA	20	77
*ldh-b*	AGATGTTGACGTTCCTCTGC	ACTCTGTGACCGCTAACTCC	20	102
*18S*	ACCCAAAGACTCTGGTTTCC	AGATACCGTCGTAGTTCCGA	20	104

The relative transcript levels of the target genes were calculated by a comparative Ct method using the 2^-ΔΔ*Ct*^ formula (Livak and Schmittgen, 2001). The relative quantification of each gene was normalized to a reference gene (*18S*) and expressed relative to a calibrator sample, where ΔΔ*C*_t_=[Δ*C*_*t, target* (sample)_ - Δ*C*_*t,18S* (sample)_] − [(Δ*C*_*t, target* (calibrator sample)_ − Δ*C*_*t,18S* (calibrator sample)_]. To validate the normalization calculations, we previously confirmed *18S* as a suitable internal control gene due its uniform efficiency and stable expression under experimental climate scenarios (one-way ANOVA, *P* > 0.05). In addition, *18S* ribosomal RNA has been used as endogenous reference gene in previous studies with Amazonian fish (Anjos *et al.*, 2011; Baptista *et al.*, 2016; Vásquez KL, 2009, PhD Thesis, Federal University of Amazonas, Manaus; Oliveira CPF, 2010, PhD Thesis, Federal University of Amazonas, Manaus). An untreated control from the baseline scenario was selected as a calibrator sample for qRT-PCR relative quantification assays.

### Enzyme activity

Absolute activities of lactate dehydrogenase (EC 1.1.1.27, L-lactato: NAD^+^ oxidoreductase) were measured at 340 nm, according to Driedzic and Almeida-Val (1996) with modifications, using a microplate reader SpectraMax Plus 384 (Molecular Devices). As the small size of tetras requires the use of a magnification lens to collect separate organs, imposing a longer time to process the samples, 48 individuals of each species were transversely divided into two portions: head (brain, heart, and liver) and tail (mostly white muscle). Each portion represents the differential contribution of the two Ldh genes: the head portion is expected to have a dominance of the *ldh-b*-like isoform, and the tail portion a dominance of the *ldh-a*-like isoform, as previously described for many other teleost species (Whitt *et al.*, 1973; Almeida-Val and Val, 1993). Both body portions (or sides) were manually homogenized in an ice-cold buffer solution containing 150 mM imidazole, 1 mM EDTA and 1% Triton X-100 (pH 7.4) in a tissue/buffer ratio 1:10 (w/v). Homogenates were centrifuged at 15,000 x *g* for 15 min at 4 °C. The assay mixture consisted of 0.15 mM NADH and 50 mM imidazole, pH 7.4 at 25 °C. All reactions were initiated by the addition of 1 mM pyruvate as the low substrate concentration (Hochachka *et al.*, 1978). The high pyruvate concentration (10 mM) was also used to measure low/high (L/H) pyruvate activity ratios as described in Bailey and Wilson (1968). L/H values were calculated as the ratio between the activity obtained with 1 mM pyruvate and that obtained with 10 mM. An L/H > 1.0 indicates Ldh inhibition (favoring aerobic metabolism and Ldh-B_4_ predominance), whereas an L/H ≤ 1.0 indicates non-inhibition of Ldh (supporting anaerobic metabolism and Ldh-A_4_ predominance). Ldh activity is expressed as μmol pyruvate·min^-1^·g wet tissue^-1^.

### Statistical analysis

Relative gene expression and Ldh activity are presented as mean ± SEM (standard errors of means; *n*=4-6). Mean differences were evaluated by two-way ANOVA with scenarios (*df*=3) and acclimatization time (*df*=1) as factors, followed by Bonferroni multiple comparisons post-hoc tests. A significant difference was assumed when *P* < 0.05. SigmaStat version 3.5 was used for statistical analysis, and graphs were built using SigmaPlot version 11.0.

## Results

### Experimental conditions

Real-time changes in temperature and CO_2_ levels in the three microcosms followed the environmental conditions in the control room over the experimental period (Figure S1). We observed no variation (one-way ANOVA; *F*=0.158, *df*=3, *P*=0.925) in air humidity between the current (61.74 ± 1.63), mild (60.80 ± 1.49), moderate (60.29 ± 1.39), and extreme (60.89 ± 1.50) scenarios.

Water quality of experimental tanks is described in Table 2. Significant increases in temperature and CO_2_ concentration in the water were observed, showing the effectiveness of the microcosm experimental setup. The minor decrease in dissolved oxygen concentrations in experimental tanks of the mild scenario did not represent a hypoxic situation for the studied species (Oliveira *et al.*, 2008; Marshall *et al.*, 2011; Campos *et al.*, 2016).

**Table 2:** Temperature, carbon dioxide, oxygen and pH of the water used for fish exposure in the microcosms (at day 30)^a^.

Emission Scenario	Temperature (°C)	CO_2_ (ppm)	Dissolved oxygen (mgL^-1^)	pH
*P. axelrodi*				
Current	27.63 ± 0.22	15.22 ± 1.57	6.96 ± 0.14	5.55 ± 0.11
	(25.1-29.3)	(5-29)	(4.62-8.53)	(4.86-8.02)
Mild	29.22 ± 0.23*	19.55 ± 1.64	6.23 ± 0.12*	5.62 ± 0.14
	(26.5-30.9)	(10,4-29)	(4.9-7.44)	(4.29-8.6)
Moderate	29.960.25*	24.21 ± 1.77	6.89 ± 0.11	5.46 ± 0.09
	(27.1-32)	(9-56)	(5.85-8.68)	(4.89-7.59)
Extreme	31.53 ± 0.19*	34.06 ± 3.22*	6.55 ± 0.10	5.22 ± 0.11
	(29.3-32.9)	(7-78)	(5.5-8.05)	(4.27-7.86)
*P. simulans*				
Current	27.80 ± 0.22	15.10 ± 1.92	6.90 ± 0.17	5.76 ± 0.11
	(24.7-29.6)	(5,5-34)	(4.87-8.61)	(4.56-7.98)
Mild	29.36 ± 0.23*	18.02 ± 1.57	5.50 ± 0.12*	5.81 ± 0.07
	(26.7-31.2)	(7-32)	(4.65-6.92)	(5.05-7.19)
Moderate	30.43 ± 0.23*	22.82 ± 1.79	6.47 ± 0.15	5.64 ± 0.08
	(27.5-32.1)	(8-35)	(4.98-8.27)	(5.21-7.78)
Extreme	32.20 ± 0.22*	33.52 ± 3.64*	6.49 ± 0.12	5.45 ± 0.10
	(29.6-33.8)	(16-60)	(5.42-7.87)	(4.32-7.62)

a Data are shown as mean ± SEM; minimum and maximum values in parenthesis. Sample size for each parameter: *n*=30 for each experimental aquarium.

* Significant differences from current scenario (one-way ANOVA, *P* < 0.05).

### Fish survival

**Figure 1:**
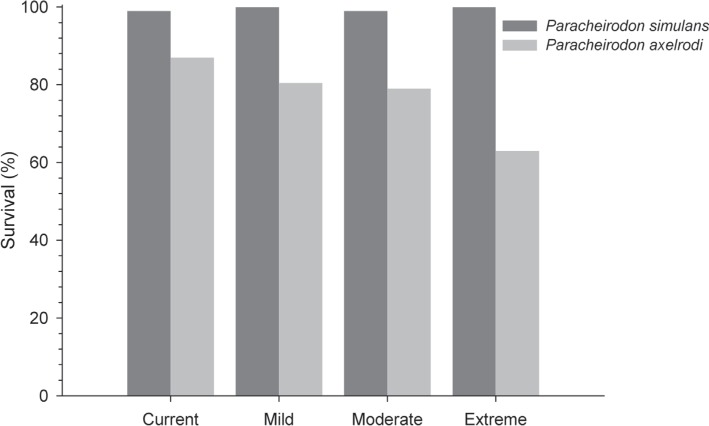
Survival (%) of *Paracheirodon axelrodi* and *P. simulans* artificially acclimatized in climate change scenarios. Fish were exposed for 30 days in current, mild, moderate and extreme scenarios simulated in the microcosms.

Survival percentages were different between the two species and among scenarios for *P. axelrodi* (Figure 1); increasing mortality was observed for *P. axelrodi* according the severity of the climate scenarios.

### Relative expression of Ldh genes

**Figure 2:**
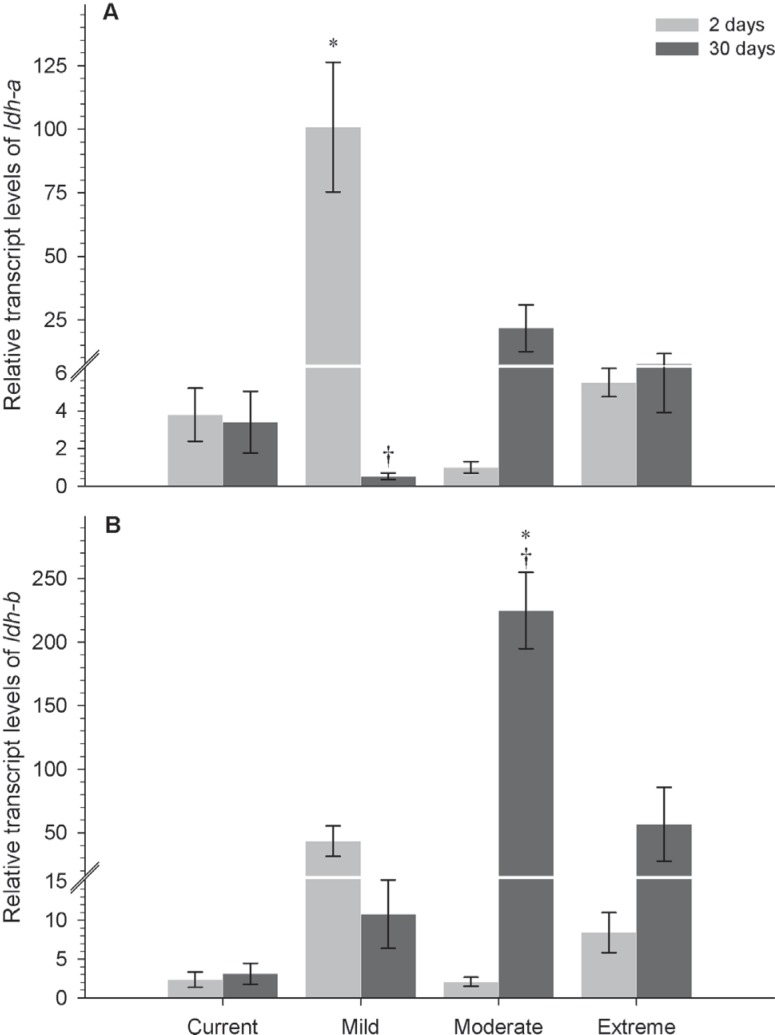
Relative expression of Ldh genes in *P. axelrodi.* Differential transcript levels of *ldh-a* (A) and *ldh-b* (B) after 2 and 30 days in current, mild, moderate and extreme climate scenarios. Data are reported as mean ± SEM. Sample sizes for both genes: *N*=4. *Significant differences from current scenario; † Significant differences between acclimatization times within a given scenario (two-way ANOVA, *P* < 0.05).

**Figure 3:**
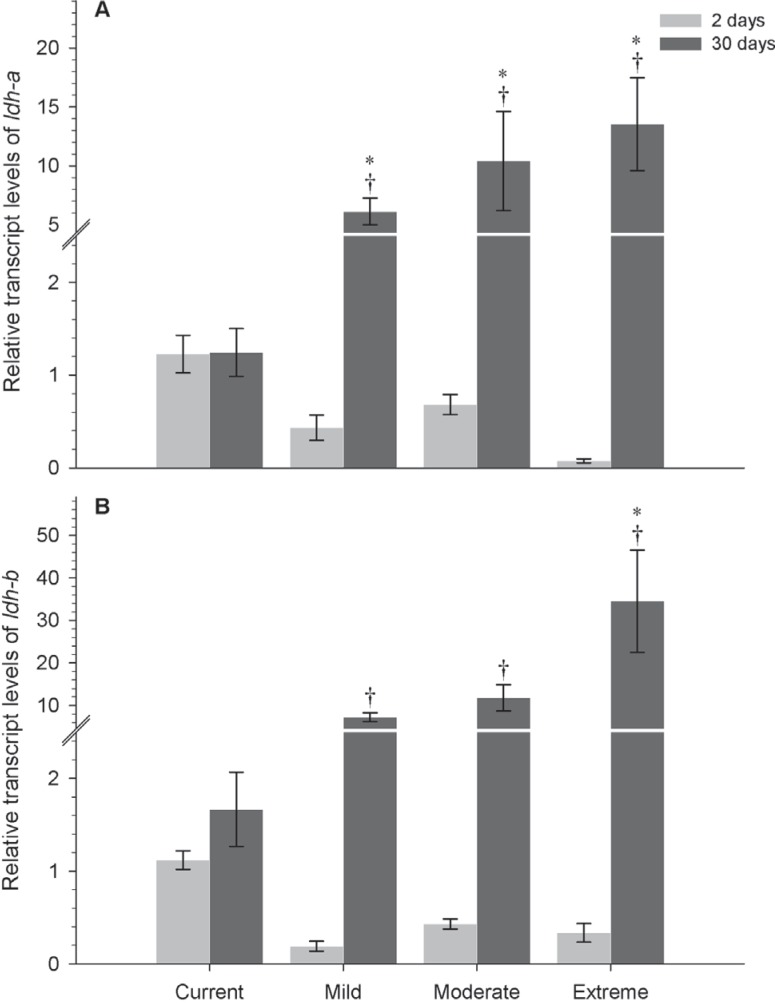
Relative expression of Ldh genes in *P. simulans.* Differential transcript levels of *ldh-a* (A) and *ldh-b* (B) after 2 and 30 days in current, mild, moderate and extreme climate scenarios. Data are reported as mean ± SEM. Sample sizes for both genes: *N*=4. *Significant differences from current scenario; † Significant differences between acclimatization times within a given scenario (two-way ANOVA, *P* < 0.05).

Anaerobic responses, measured as transcripts of *ldh-a* and *ldh-b* genes, were different in both species. While no clear correlated responses of gene transcription to climate severity were observed for *P. axelrodi* (Figure 2), *P. simulans* exhibited a direct increase of *ldh-a* and *ldh-b* transcription according to the severity of the experimental climate scenarios (Figure 3). Post-hoc comparisons showed an immediate increase in *ldh-a* transcription (approximately 25-fold) in *P. axelrodi* specimens exposed for two days to the mild scenario compared with the baseline scenario (*F*=5.016, *P* < 0.001); and a 192-fold decrease after 30 days in the mild scenario (*F*=4.855, *P* < 0.001) (Figure 2A). Furthermore, *ldh-b* mRNA levels increased 72-fold in this species when exposed for 30 days to the moderate scenario, compared to fish in baseline scenario (*F*=7.810, *P* < 0.001), and increased 108-fold compared to fish acclimated for two days under the same scenario (*F*=8.388, *P* < 0.001) (Figure 2B).

Post-hoc comparisons for *P. simulans* showed an increase in *ldh-a* transcription rates among climate scenarios and over the acclimatization periods: approximately 14-fold in mild (*F*=2.683, *P*=0.009) and moderate (*F*=4.617, *P* < 0.001) scenarios, and 180-fold in the extreme scenario (*F*=6.276, *P* < 0.001). When compared with the baseline scenario, *ldh-a* expressed as follows: 5-fold increase in the mild (*F*=3.084, *P*=0.016), 8-fold increase in the moderate (*F*=3.922, *P*=0.001), and 11-fold increase in the extreme emission scenario (*F*=5.106, *P* < 0.001) (Figure 3A). Fish exposed for 30 days to the three IPCC scenarios showed higher levels of *ldh-b* transcription compared to the base-line scenario, reaching a 20-fold increase at the extreme scenario compared with control (*F*=8.181, *P* < 0.001) and a 103-fold increase compared with fish acclimated for two days under similar conditions (*F*=9.703, *P* < 0.001) (Figure 3B).

**Figure 4:**
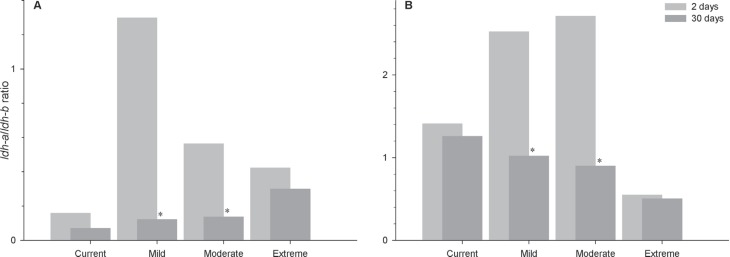
*ldh-a/ldh-b* ratios in *P. axelrodi* (A) and *P. simulans* (B) artificially acclimatized for 2 and 30 days in current, mild, moderate and extreme scenarios. All data are reported as means. Sample sizes are given in Figures 2 and 3. *Significant differences between acclimatization times within a given scenario (Student’s *t*-test, *P* < 0.05).

The ratios between the transcriptions of the two target genes (*ldh-a/ldh-b*) confirmed the quick response of these anaerobic isoform genes (Figure 4). A similar profile appeared for both species; *ldh-a/ldh-b* values rapidly increased in fish exposed for two days to all climate scenarios, subsequently stabilizing in fish exposed for 30 days, particularly at mild (Student’s t-test; *t*=3.011, *df*=19, *P*=0.007 for *P. axelrodi;* *t*=3.073, *df*=19, *P*=0.006 for *P. simulans*) and moderate (Student’s *t*-test; *t*=3.918, *df*=19, *P=* < 0.001 for *P. axelrodi;* *t*=2.461, *df*=24, *P*=0.021 for *P. simulans*) scenarios.

### Enzyme activities

Similarly to gene transcription, Ldh activities of both body portions of tetras responded to the tested climate scenarios as well as to time of artificial acclimatization (*P* < 0.05) (Table 3). *P. axelrodi* individuals exposed for two days to moderate and extreme scenarios presented, respectively, a significant increase in Ldh activity in head (*F*=3.430, *P*=0.001; *F*=2.632, *P*=0.012) and tail (*F*=5.396, *P* < 0.001; *F*=4.921, *P* < 0.001) portions when compared to the fish under the baseline scenario. After 30 days, enzyme activity in head and tail changed, respectively, in a similar way; i.e., significantly increased in fish exposed to control (*F*=2.461, *P*=0.018; *F*=3.197, *P*=0.003) and mild (*F*=0,332, *P*=0.05; *F*=2.153, *P*=0.037) scenarios, and decreased at moderate (*F*=2.966, *P*=0.005; *F*=3.437, *P*=0.001) and extreme (*F*=2.224, *P*=0.032; *F*=2.987, *P*=0.006) scenarios.

**Table 3:** Lactate dehydrogenase (Ldh) activity (1 mM pyruvate) predominant in head and tail portions of *Paracheirodon axelrodi* and *P. simulans* acclimated for two and 30 days at climate scenarios simulated in the microcosms^a^.

Emission Scenario	Head portion		Tail portion	
	2 days	30 days	2 days	30 days
*P. axelrodi*				
Current	55.97 ± 1.43	60.74 ± 1.38	58.67 ± 0.79	66.84 ± 3.36
Mild	57.47 ± 0.96	60.68 ± 1.15	59.40 ± 0.71	66.00 ± 2.22
Moderate	64.03 ± 1.20*	58.90 ± 0.63	70.78 ± 5.88*	60.37 ± 0.17
Extreme	63.32 ± 0.91*	58.99 ± 0.26	67.11 ± 1.10*	60.19 ± 0.59
*P. simulans*				
Current	47.19 ± 2.79	48.02 ± 1.20	59.70 ± 0.59	61.79 ± 3.28
Mild	46.46 ± 1.07	49.04 ± 2.31	57.70 ± 1.08	59.54 ± 0.46
Moderate	55.45 ± 1.82	50.75 ± 1.47	62.68 ± 1.08*	57.87 ± 0.43
Extreme	52.33 ± 1.32*	45.55 ± 0.35	63.83 ± 0.53	60.56 ± 0.66

a Ldh activity is reported as μmol pyruvate·min^-1^·g wet tissue^-1^ (mean ± SEM). Sample size for each tetra species: *N*=6.

* Significant differences from current scenario; Significant differences between acclimatization times within a given scenario (two-way ANOVA, *P* < 0.05).

Regarding *P. simulans*, we observed a significant increase in Ldh activity in head and tail portions of animals exposed for two days to the extreme and moderate scenarios comparing to the current one (*F*=2.163, *P*=0.037; *F*=3.443, *P*=0.001). However, fish artificially acclimatized for 30 days presented a decrease in Ldh values in the head portion in moderate scenario (*F*=2.519, *P*=0.016), and in the tail portion of fish under the extreme scenario (*F*=2.822, *P*=0.007), compared to two-day exposure.

**Figure 5:**
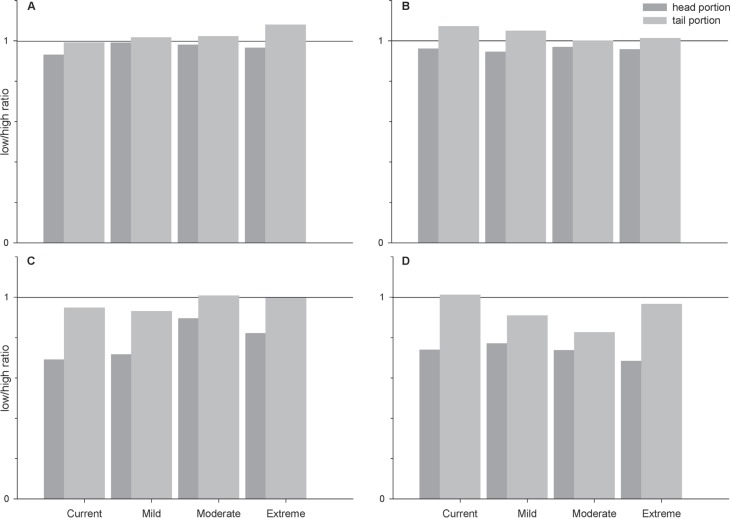
Low/high ratios in head and tail portions of both species. *P. axelrodi* (A and B) and *P. simulans* (C and D). Fish were acclimatized for two days (A and C) and 30 days (B and D) at climate scenarios. The values represent ratios and all data are reported as means.

Low/high (L/H) ratios obtained in the head and tail portions of both species after exposure to the analyzed climate scenarios are presented in Figure 5. The values were equal to, or lower than 1.0, indicating activation of Ldh at higher pyruvate concentration (10 mM) and, therefore, the increase of anaerobic glycolysis in all fish portions. These data also confirm the predominance of Ldh-A_4_ isoforms in both body portions: head portion values ranged from 0.84 to 1.08, and tail portions from 0.72 to 1.02.

## Discussion

Daily and seasonal variations of physicochemical parameters of aquatic environments such as fluctuations in temperature cause multiple physiological effects on fishes, influencing several ecological characteristics, including their natural distribution (Beitinger *et al.*, 2000; Schmidt-Nielsen, 2002). *P. axelrodi* and *P. simulans* inhabit thermally distinct habitats in the middle region of the Negro river, as observed by Marshall *et al.* (2011). The minimum and maximum daily water temperatures in their micro-habitats range from 25.1 °C to 29.9 °C for *P. axelrodi*, and from 24.6 °C to 35.2 °C for *P. simulans.* The higher water temperature in *P. simulans* natural habitats suggests a better thermal tolerance of this species to higher temperatures. Campos *et al.* (2016) described their differential thermal tolerance through the Thermal Tolerance Polygons, suggesting that they present maximum and minimum thermal limits according to their differential responses to acclimation temperatures. Thus, they suggested that *P. simulans* tolerates higher temperatures compared to *P. axelrodi.* The data herein described confirm a better ability of *P. simulans* to support higher temperatures once this species exhibited 100% survival in all three tested climate scenarios. Instead, *P. axelrodi* presented a decreased survival according to the severity of the climate scenarios, reaching only 67% at the extreme scenario (Figure 1). The adaptation to their natural environmental niches must account for their differential acclimatization at these artificial climate scenarios set as fore-seen by IPCC for the year 2100. The effective temperature in each of the species natural microenvironment resulted in different metabolic responses, including differential *P*_crit_ values, which accounts for differential hypoxia tolerance as well (Campos *et al.*, 2016). The differences found in survival responses are just a glance (30-day exposure) of what may occur in the many generations until the year 2100. However, the data suggest that mortality may occur shortly if fish do not adapt through gene plasticity (epigenetic changes, for instance) or selection of favoring mutation in enzymes/proteins. Furthermore, the velocity in which the changes are occurring could be a severe threat to all living species, since evolutionary responses and adaptation to an environmental change might require a much longer time range.

Previous studies addressing lethal temperatures of *P. axelrodi* (LT_50_, 96 h = 33.7 °C) resulted in 100% fish survival between 29 and 31 °C, reaching total fish mortality at temperatures above 35 °C (Oliveira *et al.*, 2008). The recent study by Campos *et al.* (2016) described the minimum and maximum thermal limits for both species, using critical thermal methodology (CTM): 13 to 40 °C for *P. axelrodi* and 12 to 42 °C for *P. simulans.* Thus, as we observed in the present work, *P. simulans* tolerated a wider temperature range than *P. axelrodi.* Prodocimo and Freire (2001) reported similar results for the tropical platyfish *Xiphophorus maculatus*, an ornamental freshwater teleost. They determined the thermal tolerance limits ranging from a minimum of 9.6 °C to a maximum of 41.5 °C, showing the greater capacity of tropical species to withstand high temperatures better than low ones.

Herein, both *P. axelrodi* and *P. simulans* adapted to high temperatures (Axelrod *et al.*, 1986), although *P. simulans* tolerated a higher range compared to *P. axelrodi*. As mentioned, Campos *et al.* (2016) demonstrated that *P. simulans* is also more tolerant to hypoxia, indirectly measured by critical oxygen tension (*P*_crit_), and exhibits higher metabolic rates than *P. axelrodi*, what explains its greater ability to tolerate high temperatures. A recent study by McBryan *et al.* (2016) showed that warm acclimation of two *Fundulus heteroclitus* subspecies (southern-warm and northern-cold) improves their hypoxia tolerance. They also showed different measurements of LOE_hyp_ (loss of equilibrium in hypoxia), critical oxygen tension (*P*_crit_) and total lamellar surface area in warm acclimated fish. Warm acclimation caused different hypoxia tolerance levels between the two subspecies suggesting that the differences are related to specific metabolic rates, as southern subspecies (acclimatized to the warmer environment) are more tolerant to increasing temperatures than the northern subspecies (acclimatized to the colder environment). The authors concluded that the two subspecies have unique plasticity and adaptation processes acting on the oxygen cascade. In accordance to that study and to Campos *et al.* (2016), this study found different mortality percentages between the two species, although the concept of phenotypic plasticity cannot be applied to explain the results, since different species were used. To better understand these differences, the anaerobic capacity of these two species was tested in the present work throughout the relative measurement of Ldh genes transcription and enzyme activities. Heuton *et al.* (2015) have shown that desert pupfish (*Cyprinodon diabolis*) that were acclimated differently to ecologically suitable temperatures exhibited periods of anaerobiosis when exposed to increasing temperatures, despite oxygen availability.

Acclimation and adaptation processes of organisms facing environmental oxygen or temperature changes involve two basic adjustments: (i) quantitative variations in genes expression by suppression or induction of control mechanisms, and (ii) qualitative changes affecting the production of alternative isoforms that favor adaptive changes (Hochachka and Somero, 2002). These quantitative changes reflect molecular mechanisms for the reorganization of metabolism that significantly contributes to the adaptive responses to abiotic environmental variations (Schulte, 2004). In the present study, the short- (two days) and long-term (30 days) exposure to future climate scenarios induced differential transcription of *ldha-a* and *ldh-b* genes in *P. axelrodi* and *P. simulans*. We found that *P. axelrodi* survival was more affected than *P. simulans* when temperature was synergistically associated with carbon dioxide in the climate scenarios. We observed a significant increase in *ldh-a* mRNA transcription in *P. axelrodi* after two days (acute exposure) in mild emission scenario (Figure 2A), suggesting that this gene plays a significant role in the activation of anaerobic metabolism, providing rapid responses to temperature changes due to the sudden increase in fish energy demand (Almeida-Val *et al.*, 2006). Seasonal variations of the *ldh-a* and *ldh-b* gene expression products were previously described in the tropical fish tambaqui (*Colossoma macropomum*) in their natural environment; variations were related to fluctuations of both temperature and dissolved oxygen (Almeida-Val *et al.*, 1990). The increase of *ldh-a* product in both heart and skeletal muscle of *C. macropomum*, along with the loss of *ldh-b* product in heart tissue indicated the increase in anaerobic power during acclimatization. A similar trend occurred in the present study with *P. axelrodi* when considering the increase of *ldh-a* transcripts.

The artificial acclimatization of both species for 30 days to increased temperature and CO_2_ levels caused adjustments of Ldh genes transcription. When exposed to the mild and moderate climate scenarios, specimens of *P. axelrodi* presented a decrease in the *ldh-a* transcription and an accentuated increase in the *ldh-b* transcription, respectively. Thus, this species does not rely entirely on anaerobic metabolism, requiring the contribution of *ldh-b* gene, which is predominantly active in aerobic tissues (Figure 2A,B). Crawford and Powers (1992) observed an increase of *ldh-b* mRNA in liver of killifish (*Fundulus heteroclitus*) acclimated to high temperatures. These changes result in increased survival (Powers and Schulte, 1998) and increased ability of tropical fishes in dealing with high temperatures in their natural environment (Edmunds *et al.*, 2009). *P. simulans*, instead, increased Ldh gene transcripts after long-term experimental acclimatization (30 days) to the three scenarios (mild, moderate and extreme) (Figure 3A, B). Thus, *P. simulans* relies on anaerobic metabolism to survive higher metabolic demands. Wootton (1990) suggested that the biochemical and physiological responses depend on the time scale of the environmental changes. If the changes persist, fishes may acclimatize using adjustments in gene expression (phenotypic plasticity) to maintain its homeostasis (Almeida-Val *et al.*, 1999). In this study, these changes in gene transcription reflected the Ldh activity levels.

Analysis of total Ldh activities (Table 3) revealed that after two days of exposure, head portions of both *P. axelrodi* and *P. simulans* had increased Ldh in moderate and extreme scenarios, suggesting an activation of anaerobic metabolism in tissues where Ldh-B_4_ isoform is predominant. According to Chippari-Gomes *et al.* (2005), the increase in Ldh levels with a concomitant decrease in citrate synthase (CS) in heart tissue of two Amazonian cichlids (*Astronotus crassipinis* and *Symphysodon aequifasciatus*) exposed to hypoxia and anoxia indicates the anaerobic potential of this tissue due to the accumulation of pyruvate and the concomitant decrease in aerobic metabolism. A similar trend occurred in tail portions (where *ldh-a* gene predominates) of the species herein analyzed, resulting in increased Ldh activities in the same moderate and extreme climate scenarios. Davies *et al.* (2011) observed changes in Ldh-A isoform activities in white muscle of the bluegill (*Lepomis macrochirus*) and the pumpkinseed (*Lepomis gibbosus*) after exposure to acute hypoxia. Pumpkinseed is a hypoxiatolerant species, which showed elevated activities of Ldh as well as higher transcript levels of *ldh-a* mRNA compared to bluegill. The similarity of metabolic responses between animals exposed to hypoxia or high temperature reflect energy requirements of the cell due to lower oxygen availability in the cellular milieu. After 30 days, *P. axelrodi* and *P. simulans* reached a stabilized condition for Ldh in both body portions. Compared to the two-day exposure, a significant increase of anaerobic potential occurred in both parts of *P. axelrodi* acclimated to the current and mild scenarios, followed by a concomitant reduction in the moderate and extreme scenarios, suggesting a metabolic suppression in these conditions. Differently, for *P. simulans*, significant decreases in anaerobic metabolism in the head portion (moderate scenario) and tail portion (extreme scenario) were observed.

Acclimatization responses to environment challenges (in the present work, synergistic effects of elevated temperature and CO_2_ levels) often modulate the activities of metabolic enzymes (Crawford, 2002; Chippari-Gomes *et al.*, 2005). Furthermore, Ldh activities can be altered by substrate concentration, temperature, oxygen, and pH (Almeida-Val *et al.*, 1991). A recent review by Storey (2016) suggests that Ldh also changes its phosphorylation state as a stress-induced response in several organisms, including fishes. This post-translational modification (PTM) of Ldh leads to substantial changes in enzyme properties, so that the phosphorylated form is inhibited compared to the non-phosphorylated form. The changes of Ldh absolute activities in the two analyzed species suggest that post-translational changes, such as reversible protein phosphorylation or other epigenetic change, may explain the balance between anaerobic and aerobic metabolisms, helping fish to face different climate scenarios (Figure S2). Herein, Ldh measured with high pyruvate concentration (10 mM, an inhibitory concentration for most fish tissues) presented increased values in head and tail portions of both species (Table S2), reflecting the absence of pyruvate inhibition in all experimental scenarios, i.e., increased anaerobic metabolism. These L/H ratios confirm gene predominance for Ldh and indicate which type of metabolism (aerobic or anaerobic) is predominant in the tissues (Almeida-Val and Val, 1990). The two studied species displayed low or no inhibition ratios for Ldh at the two body parts (Figure 5). Either portion may contain higher amounts of skeletal muscle isoform, i.e., both portions must have more Ldh-A polypeptides than Ldh-B polypeptides, which can be seen in the ratios observed in Figure 4 regarding the overexpression of *ldh-a* gene over *ldh-b* in the whole body of these animals when exposed to all climate scenarios. In fact, L/H ratios are lower in skeletal muscle as already described for many species (Almeida-Val and Val, 1990; Almeida-Val *et al.*, 1991, 1995; Almeida-Val and Farias, 1996). Ldh-A_4_ orthologues are not inhibited by 10 mM pyruvate, indicating the predominance of anaerobic metabolism in muscle tissues, which is expected especially in the tail portion of both species. As known, both species have a predominance of isozyme Ldh-A_4_ in the whole body, with a significant decrease of Ldh-B_4_ isozyme in tail portion.

Long-term exposure to the extreme climate scenario may have induced an artificial acclimatization in both species, helping fish deal with environmental changes, although the species *P. simulans* can be considered better adapted than *P. axelrodi.* Also, *P. simulans* showed a higher ability to regulate Ldh gene transcription during short- and long-term exposure, which should help this species to better survive climate scenarios predicted for the year 2100 using its anaerobic power. In contrast, *P. axelrodi* was unable to regulate its *ldh-a* and *ldh-b* mRNA during such period, suggesting that the effects of climate change on tropical teleosts, particularly on fish of the Amazon, cannot be generalized.

Overall, Ldh gene regulation in these species leads to the predominance of anaerobic glycolysis in fish exposed to environmental climate change. We suggest that post-translational modifications can also regulate protein kinetic properties to allow survival of these species; further studies are encouraged. In conclusion, these results reflect the particular adaptive characteristics that each species develops during the evolutionary line to cope with temperature changes in its own habitat, and how differently these congeneric species will be affected by the ongoing climate-driven environmental changes.

## Supplementary material

The following online material is available for this article:

Figure S1 Environmental conditions of the microcosms in 30 days of experiment.

Figure S2 Pearson’s correlations for Ldh relative gene expression and enzyme activity.

Table S1 Partial sequences of the *ldh-a, ldh-b* and *18S* genes.

Table S2 Lactate dehydrogenase (Ldh) activity in head and tail portions.
