# Supplementary material for: Differential survivorship of congeneric ornamental fishes under forecasted climate changes are related to anaerobic potential
Source: Genet Mol Biol. 2018 Feb 19;41(1):107–18. doi: 10.1590/1678-4685-GMB-2017-0016 (PMC5901506; doi:10.1590/1678-4685-GMB-2017-0016)
Supplement: Supplementary file 1 [file 1415-4757-gmb-1678-4685-GMB-2017-0016-Suppl03.pdf]

Supplementary material to “Differential survivorship of congeneric ornamental fishes under forecasted climate changes are related to anaerobic potential”

Table S1 - Partial sequences of the *ldh-a*, *ldh-b* and *18S* genes in *P. axelrodi* (A) and *P. simulans* (B), respectively.

(A)

ldh-a partial sequence (463 pb)
GCCCGTCAGCAGGAGGGCGAGAGCAGGCTCAACCTTGTGCAGAGGAACGTCAACATCTTCAAGCACA
TCATCCCTCAGATCGTCAAGTACAGCCCTAACTGCACCCTTGTTGTGGTGTCTAACCCAGTGGATGTCT
TGACCTACGTCACCTGGAAGTTGAGTGGCCTGCCCAAGCACCGTGTCATTGGCAGTGGCACCAACCTG
GACTCTGCCCGCTTCCGCTACATCATGGCTGAGAAGCTGGGCATCCATGCCACCAGCTTTAACGGCTA
CATCTTGGGAGAGCACGGAGATTTCAGTGTGCCTGTATGGAGTGGTGCTAACGTTGCTGGAGTTAGCC
TGCAGAAACTCAACCCCGCCATCGGCACTGATCAGGACAGCGAGAACTGGAAGGAGGCTCACAAGAT
GGTGGTGGACAGTGCTTATGAGGTCATCAAGCTGAAGGGCTACACCTCCTGGGCCA
ldh-b partial sequence (301 pb)
GACGATCTGAGGGATGATGTGCTTGAAGATGTTGACGTTCCTCTGCACAAGGTTGAGCCTGCTCTCGC
CCTCCTGCTGGCGTACACCCGCGGTCACAACCACGATGCGGGAGTTAGCGGTAACAGAGTAGTCCTTG
TCAGCCACGATCTTGTGCGTCTTGAGGAAAAGGCTGCCATGCTGCAGGTCCATCATCTCTCCCTTCAGC
TTGTCTTCAATCACTATCAACCAGAGCGAGCTCATCACACAAGTCCCTGAGCAAAATGCTGACGGCAC
AGGCCATGCCCACTTGCCCCACTCCCAA
18S partial sequence (204 pb)
TTTTTAAGTTTCAGCTTTGCAACCATACTCCCCCCGGAACCCAAAGACTCTGGTTTCCCCGCACGCTGC
CCGGCGGGTCATGGGAATAACCCGCCGCATCGCGGGTCGGCATAGTTTACGGTCGGAACTACGACGG
TATCTGATCGTCTGGCGAACCTCCGACTTTCGTTCTTGATTAATGAAAACATTCTTGGCAAATGCTTA

(B)

ldh-a partial sequence (465 pb)
GCCCGCCAGCAGGAGGGCGAGAGCAGGCTCAACCTTGTGCAGAGGAACGTCAACATCTTCAAGCACA
TCATCCCTCAGATCGTCAAGTACAGCCCTAACTGCACCCTTGTTGTGGTGTCTAACCCAGTGGATGTCT
TGACCTACGTGACGTGGAAGTTGAGTGGCCTGCCCAAGCACCGTGTCATTGGCAGTGGCACCAACCTG
GACTCTGCCCGCTTCCGCTACATCATGGCCGAGAAGCTGGGCATCCATGCCACCAGCTTTAACGGGTA
CATCTTGGGAGAGCACGGAGACTCCAGTGTGCCTGTATGGAGTGGTGCTAACGTTGCTGGAGTTAGCC
TTGCAGAAACTCAACCCCGCCATCGGCACTGATCAGGACAGTGAGAACTGGAAGGAGGCTCACAAGA
TGGTGGTGGACAGTGCTTATGAGGTGATCAAGCTGAAGGGCTACACCTCCTTGGGCCA
ldh-b partial sequence (309 pb)
ACTGTGTTTGGCGATCTGAGGGATGATGTGCTTGAAGATGTTGACGTTCCTCTGCACAAGGTTGAGCC
TGCTCTCGCCCTCCTGCTGGCGTACACCCGCGGTCACCACCACGATGCGGGAGTTAGCGGTCACAGAG
TAGTCCTTGTCAGCCACGATCTTGTGCGTCTTGAGGAAAAGGCTGCCATGCTGCAGGTCCATCATCTCT
CCCTTCAGCTTGTCTTCAATCACATCAACCAGAGCGAGCTCATCACACAGGTCCCTGAGCAAAATGCT
GACGGCACAGGCCATGCCCACTTGCCCCACTCCCAA
18S partial sequence (203 pb)
TTTAAGTTTCAGCTTTGCAACCATACTCCCCCCGGAACCCAAAGACTCTGGTTTCCCCGCACGCTGCCC
GGCGGGTCATGGGAATAACGCCGCCGGATCGCGGGTCGGCATAGTTTACGGTCGGAACTACGACGGT
ATCTGATCGTCTTCGAACCTCCGACTTTCGTTCTTGATTAATGAAAACATTCTTGGCAAATGCTTAA
