# Supplementary material for: Differential survivorship of congeneric ornamental fishes under forecasted climate changes are related to anaerobic potential
Source: Genet Mol Biol. 2018 Feb 19;41(1):107–18. doi: 10.1590/1678-4685-GMB-2017-0016 (PMC5901506; doi:10.1590/1678-4685-GMB-2017-0016)
Supplement: Supplementary file 2 [file 1415-4757-gmb-1678-4685-GMB-2017-0016-Suppl01.pdf]

Supplementary material to "Differential survivorship of congeneric ornamental fishes under forecasted climate changes are related to anaerobic potential"

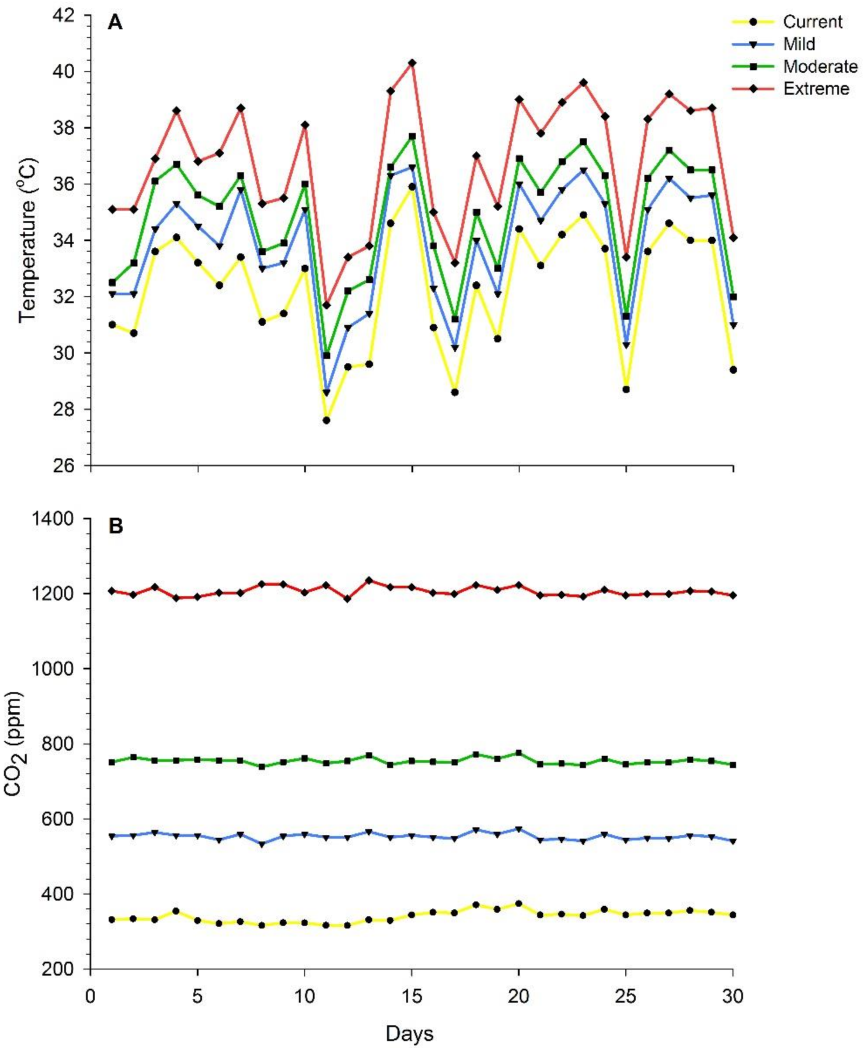

Figure S1 - Environmental conditions of the microcosms in 30 days of experiment. Temperature (A) and CO_2 (B) levels in the current, mild, moderate and extreme scenarios as forecasted by IPCC for the year 2100.
