# Supplementary material for: Differential survivorship of congeneric ornamental fishes under forecasted climate changes are related to anaerobic potential
Source: Genet Mol Biol. 2018 Feb 19;41(1):107–18. doi: 10.1590/1678-4685-GMB-2017-0016 (PMC5901506; doi:10.1590/1678-4685-GMB-2017-0016)
Supplement: Supplementary file 3 [file 1415-4757-gmb-1678-4685-GMB-2017-0016-Suppl02.pdf]

Supplementary material to "Differential survivorship of congeneric ornamental fishes under forecasted climate changes are related to anaerobic potential"

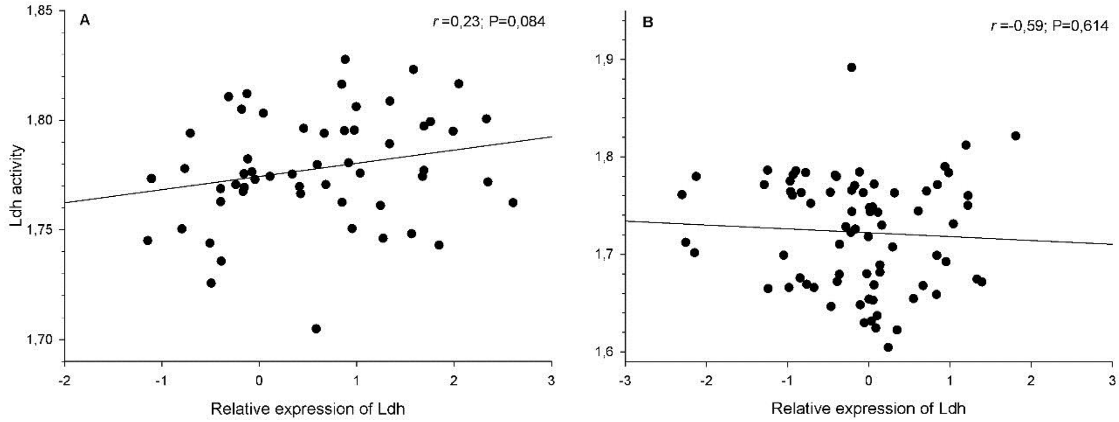

Figure S2 - Pearson's correlations between Ldh relative gene expression and enzyme activity levels in *P. axelrodi* (A) and *P. simulans* (B). Data are shown as Log_{10}.
