# Supplementary material for: Differential survivorship of congeneric ornamental fishes under forecasted climate changes are related to anaerobic potential
Source: Genet Mol Biol. 2018 Feb 19;41(1):107–18. doi: 10.1590/1678-4685-GMB-2017-0016 (PMC5901506; doi:10.1590/1678-4685-GMB-2017-0016)
Supplement: Supplementary file 4 [file 1415-4757-gmb-1678-4685-GMB-2017-0016-Suppl04.pdf]

Supplementary material to "Differential survivorship of congeneric ornamental fishes under forecasted climate changes are related to anaerobic potential"

Table S2 - Lactate dehydrogenase (Ldh) activity (10 mM pyruvate) predominant in head and tail portions of *Paracheirodon axelrodi* and *P. simulans* acclimated for two and 30 days at climate scenarios simulated in the microcosms[a].

Emission Scenario	Head portion		Tail portion	
	2 days	30 days	2 days	30 days
P. axelrodi				
Current	60.2±0.658	63.2±0.874†	59.1±0.826	62.3±1.360†
Mild	58.0±0.394	64.2±0.580†	58.3±0.521	62.9±0.802†
Moderate	65.3±0.584*	60.8±0.220*†	63.4±0.642*	60.3±0.882†
Extreme	65.6±0.786*	61.6±0.716†	62.1±0.325	59.3±0.589*†
P. simulans				
Current	68.3±0.664	65.0±0.471†	63.1±1.291	61.0±0.721
Mild	64.9±0.354*	63.7±0.280	62.1±0.431	65.5±1.098*†
Moderate	62.1±1.375*	68.9±0.722*†	62.3±1.251	70.0±0.272*†
Extreme	63.8±0.525*	66.7±0.657†	64.2±1.701	62.7±0.334

[a]Ldh activity is reported as μmol pyr·min^{-1}·gwt^{-1} (mean±SEM). Sample size for each tetra species: *N*=6. *Significant differences from current scenario; †Significant differences between acclimatization times within a given scenario (two-way ANOVA, $P<0.05$).
